# Research on the 3D Reverse Time Migration Technique for Internal Defects Imaging and Sensor Settings of Pressure Pipelines

**DOI:** 10.3390/s23218742

**Published:** 2023-10-26

**Authors:** Daicheng Peng, Xiaoyu She, Yunpeng Zheng, Yongjie Tang, Zhuo Fan, Guang Hu

**Affiliations:** 1 Key Laboratory of Exploration Technologies for Oil and Gas Resource, Yangtze University, Ministry of Education, Wuhan 430100, China; shexiaoyu2000@163.com; 2 Research and Development Center, Bureau of Geophysical Prospecting Inc., China National Petroleum Corporation, Zhuozhou 072751, China; zhengyunpeng@cnpc.com.cn; 3 Department of Earth and Space Sciences, Southern University of Science and Technology, Shenzhen 518055, China; tyj_cug@126.com; 4 Hubei Subsurface Multi-Scale Imaging Key Laboratory, School of Geophysics and Geomatics, China University of Geosciences, Wuhan 430074, China; zhuo@cug.edu.cn; 5 Southern Marine Science and Engineering Guangdong Laboratory, Guangzhou 511458, China; huguang@gmlab.ac.cn

**Keywords:** reverse time migration, 3D ultrasonic imaging, sensor settings, observation system, pressure pipelines

## Abstract

Although pressure pipelines serve as a secure and energy-efficient means of transporting oil, gas, and chemicals, they are susceptible to fatigue cracks over extended periods of cyclic loading due to the challenging operational conditions. Their quality and efficiency directly affect the safe operation of the project. Therefore, a thorough and precise characterization approach towards pressure pipelines can proactively mitigate safety risks and yield substantial economic and societal benefits. At present, the current mainstream 2D ultrasound imaging technology faces challenges in fully visualizing the internal defects and topography of pressure pipelines. Reverse time migration (RTM), widely employed in geophysical exploration, has the capability to visualize intricate geological structures. In this paper, we introduced the RTM into the realm of ultrasonic non-destructive testing, and proposed a 3D ultrasonic RTM imaging method for internal defects and sensor settings of pressure pipelines. To accurately simulate the extrapolation of wave field in 3D pressure pipelines, we set the absorbing boundary and double free boundary in cylindrical coordinates. Subsequently, using the 3D ultrasonic RTM approach, we attained higher-precision 3D imaging of internal defects in the pressure pipelines through suppressing imaging artifacts. By comparing and analyzing the imaging results of different sensor settings, the design of the observation system is optimized to provide a basis for the imaging and interpretation of actual data. Both simulations and actual field data demonstrate that our approach delivers top-notch 3D imaging of pipeline defects (with an imaging range accuracy up to 97.85%). This method takes into consideration the complexities of multiple scattering and mode conversions occurring at the base of the defects as well as the optimal sensor settings.

## 1. Introduction

Transportation by pipeline is known as a “lifeline”. Pressure pipelines refers to the use of certain pressure to transport gas or liquid via tubular equipment, a technique widely used in many industrial fields such as oil, gas, chemical, and metallurgical engineering. Due to long term exposure to high temperature and pressure, as well as corrosive agents, the performance of the pipeline is adversely affected [1]. Cracks, inclusions, thinning, and other defects may occur under long-term cyclic loading, ultimately leading to equipment failings often associated with casualties and significant economic losses [2]. Therefore, conducting a thorough, high-precision inspection of pressure pipelines, coupled with swift and precise detection of diverse defects, has the potential to proactively mitigate safety risks and yield positive economic and societal impacts [3].

In order to inspect pipelines, different approaches have been proposed, such as magnetic particle testing (MT) [4], eddy current testing (ET) [5], acoustic wave testing (AT) [6], radiographic testing (RT) [7] and, penetration testing (PT) [8]. Among the commonly applied non-destructive testing methods, MT and PT can only be used for surface crack detection [9,10]. ET can only detect surface or subsurface defects [11]. RT is harmful to the human body, and the maintenance cost is high [12]. However, it proves advantageous for discerning the structure and configuration of internal defects within pressure pipelines. Its merits include high sensitivity, compact equipment, and a non-invasive nature that poses no risks to both human health and environmental quality [13]. UT can proficiently ascertain flaw dimensions, identify crack locations [14], delamination location, and determine the stacking sequence of composites [15,16,17] through the propagation of surface waves, guided waves, and body waves [18,19]. Subsequently, employing ultrasonic imaging methods allows for the intuitive visualization of defects within pressure pipelines [20,21,22].

Conventional ultrasonic methods of pipeline testing encompass the Total Focusing Method (TFM) [23,24,25], the Synthetic Aperture Focusing Technique (SAFT) [26,27,28], and Time of Flight Diffraction (TOFD) [29,30,31]. There are pros and cons to each method. The TFM is an imaging technique that relies on data acquired through full-matrix capture (FMC) [32]. However, this method only takes into account the straight-line path of ultrasound waves, discarding the multiple scattering and mode conversion caused by interactions between ultrasonic waves and defects. Consequently, imaging structures with vertical boundaries or intricate geometry becomes challenging, and the deeper the defect in pressure pipelines the lower the imaging precision will be [33,34]. Currently, in most cases, SAFT, which is closely associated with Kirchhoff migration, is employed for imaging purposes. Nevertheless, this approach encounters challenges when attempting to image sharply inclined interfaces and the lower boundaries of tubes, holes, or similar objects. Furthermore, SAFT generated images may include artifacts caused by surface waves and multiple reflections at the interfaces, leading to incorrect conclusions regarding the locations of internal defects [35]. The use of complete acoustic or elastic wave equation simulation in modern UT may overcome these limitations. Herein, we present a method for the 3D ultrasonic imaging of pipelines using RTM which has undergone comprehensive examination in geophysics and various other disciplines [36,37,38,39,40]. During the past decade, the RTM method, originally derived from seismic imaging in geophysics, has been applied in the field of UT with promising results [41,42,43,44,45,46]. Compared to conventional ultrasonic imaging methods, the RTM stands out as a seismic processing method founded on full-wave extrapolation. This approach encompasses information about mode switching and various wave reflections, enabling the generation of high-resolution images that effectively capture internal defects characterized by complex structures [47,48].

In order to rectify the limitations inherent in conventional 2D methods and enhance the UT precision of internal defects in pressure pipelines, a 3D ultrasonic RTM imaging method of internal defects is proposed, and the sensor settings research according to the cavity cylindrical characteristics of pressure pipelines. Herein, the simulation of the 3D elastic wave reverse time extrapolation of the pressure pipelines is achieved by establishing a dual free boundary and employing an absorption boundary. Subsequently, the ultra-sonic RTM method is utilized to attain top-notch 3D imaging of defects within pipelines through the implementation of a method for imaging artifact suppression. In addition, by comparing and analyzing the imaging results in different sensor settings, the design of the observation system is optimized to provide a basis for the imaging and interpretation of actual test data. We demonstrate the reliability and effectiveness of our method by presenting numerical simulation examples and field test data, laying the foundation for practical applications in UT.

## 2. Materials and Methods

To conduct precise 3D imaging of internal defects in pressure pipelines, we have established a 3D RTM method for internal defect visualization using cylindrical coordinates. In the subsequent sections, we offer a concise overview of the reverse time extrapolation of the elastic wave field in cylindrical coordinates, boundary conditions, imaging condition, and RTM artifacts.

### 2.1. Extrapolation of the Wave-Field in Cylindrical Coordinates

In the cylindrical coordinates of an isotropic medium, the first-order velocity–stress equation is expressed as follows [49]:

(1) $$\begin{array}{l} \rho \partial_{t}v=\left[U^{\left(r\right)}+\frac{1}{r}U^{\left(\theta \right)}+U^{\left(z\right)}\right]\tau +f\\ \partial_{t}\tau =\left[V^{\left(r\right)}+\frac{1}{r}V^{\left(\theta \right)}+V^{\left(z\right)}\right]v+g \end{array}$$

where $v={\left[v_{r},v_{\theta },v_{z}\right]}^{T}$ and $\tau ={\left[\tau_{rr},\tau_{\theta \theta },\tau_{zz},\tau_{r\theta },\tau_{\theta z},\tau_{rz}\right]}^{T}$ represent the velocity and stress vectors, $f={\left[f_{r},f_{\theta },f_{z}\right]}^{T}$ denotes the point force source, and $g={\left[g_{rr},g_{\theta \theta },g_{zz},g_{r\theta },,g_{rz},,g_{\theta z}\right]}^{T}$ represents the stress force. To simulate the wave field in the time-domain finite difference method (TFDM), Equation (1) can be discretized utilizing a central differencing scheme on a staggered grid.

### 2.2. Boundary Conditions

**Figure 1:**
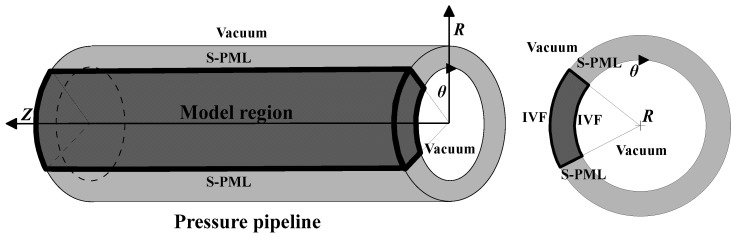
Diagram of the 3D pressure pipeline model, depicting the setting of the boundary conditions.

For proper simulation of the ultrasonic waves’ propagation in pressure pipelines, the setting of boundary conditions is particularly important when performing reverse time extrapolation. Figure 1 depicts a schematic figure of the boundary condition setup. To mitigate interference from the surrounding area beyond the model, an absorbing boundary condition is applied to the exterior. The split-field perfectly matched layer (S-PML) [50] was employed to enhance absorption while minimizing computational costs; Figure 1 illustrates the placement of S-PML in both the Z-path and R-path of the pipeline. This configuration is designed to effectively absorb body waves as they propagate towards the model boundary.

**Figure 2:**
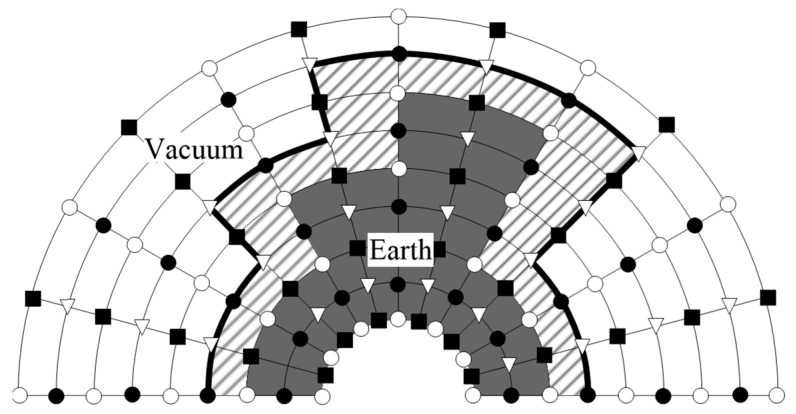
Grid distribution of the improved vacuum formulation in cylindrical coordinates. ○ and ∇ represent the different positions of normal stress wave field, model physical parameters, and shear stress wave field, respectively. 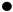 and 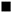 denotes the differential position of the velocity wave field.

As for the setting of free boundaries, to ensure the efficiency of computation, we introduce an improved vacuum formula (IVF) [51] and apply it in the cylindrical coordinates to establish the dual free boundaries conditions in the θ-path. The IVF proves to be more versatile when dealing with ordinary free boundaries, this stands in contrast to the acoustic–elastic boundary method, which necessitates the manual configuration of the free boundaries’ condition for each specific case. The black solid line in Figure 2 indicates the position of the free boundaries in the actual calculation, the shaded area is a virtual layer whose thickness is half of the mesh, the grey area indicates the solid medium, and the white area is the vacuum layer. The formula of the improved vacuum method in cylindrical coordinates is given by:

(2) $${\left(\rho_{r}\right)}_{i,j,k}=\{\begin{array}{cc} 0, & if\;{\left(\rho \right)}_{i+\frac{1}{2},j,k}=0\;and\;{\left(\rho \right)}_{i-\frac{1}{2},j,k}=0\\ \frac{1}{2}\left[{\left(\rho \right)}_{i+\frac{1}{2},j,k}+{\left(\rho \right)}_{i-\frac{1}{2},j,k}\right], & otherwise \end{array}$$

(3) $${\left(\rho_{\theta }\right)}_{i+\frac{1}{2},j+\frac{1}{2},k}=\{\begin{array}{cc} 0, & if\;{\left(\rho \right)}_{i+\frac{1}{2},j,k}=0\;and\;{\left(\rho \right)}_{i+\frac{1}{2},j+1,k}=0\\ \frac{1}{2}\left[{\left(\rho \right)}_{i+\frac{1}{2},j,k}+{\left(\rho \right)}_{i+\frac{1}{2},j+1,k}\right], & otherwise \end{array}$$

(4) $${\left(\rho_{z}\right)}_{i+\frac{1}{2},j,k+\frac{1}{2}}=\{\begin{array}{cc} 0, & if{\left(\rho \right)}_{i+\frac{1}{2},j,k}=0\;and\;{\left(\rho \right)}_{i+\frac{1}{2},j,k+1}=0\\ \frac{1}{2}\left[{\left(\rho \right)}_{i+\frac{1}{2},j,k}+{\left(\rho \right)}_{i+\frac{1}{2},j,k+1}\right], & otherwise \end{array}$$

(5) $$\begin{array}{ll} {\left(\mu_{r\theta }\right)}_{i,j+\frac{1}{2},k}= & \{\begin{array}{c} {\left[\frac{1}{4}\left[\frac{1}{{\left(\mu \right)}_{i+\frac{1}{2},j,k}}+\frac{1}{{\left(\mu \right)}_{i+\frac{1}{2},j+1,k}}+\frac{1}{{\left(\mu \right)}_{i-\frac{1}{2},j,k}}+\frac{1}{{\left(\mu \right)}_{i-\frac{1}{2},j+1,k}}\right]\right]}^{-1},\\ 0, \end{array}\\ & \{\begin{array}{c} if\;{\left(\mu \right)}_{i+\frac{1}{2},j,k},{\left(\mu \right)}_{i+\frac{1}{2},j+1,k},{\left(\mu \right)}_{i-\frac{1}{2},j,k},{\left(\mu \right)}_{i-\frac{1}{2},j+1,k}\neq 0\\ otherwise \end{array} \end{array}$$

(6) $$\begin{array}{ll} {\left(\mu_{rz}\right)}_{i,j,k+\frac{1}{2}}= & \{\begin{array}{c} {\left[\frac{1}{4}\left[\frac{1}{{\left(\mu \right)}_{i+\frac{1}{2},j,k}}+\frac{1}{{\left(\mu \right)}_{i+\frac{1}{2},j,k+1}}+\frac{1}{{\left(\mu \right)}_{i-\frac{1}{2},j,k+1}}+\frac{1}{{\left(\mu \right)}_{i-\frac{1}{2},j,k}}\right]\right]}^{-1},\\ 0, \end{array}\\ & \{\begin{array}{c} if\;{\left(\mu \right)}_{i+\frac{1}{2},j,k},{\left(\mu \right)}_{i+\frac{1}{2},j,k+1},{\left(\mu \right)}_{i-\frac{1}{2},j,k+1},{\left(\mu \right)}_{i-\frac{1}{2},j,k}\neq 0\\ otherwise \end{array} \end{array}$$

(7) $$\begin{array}{ll} {\left(\mu_{\theta z}\right)}_{i+\frac{1}{2},j+\frac{1}{2},k+\frac{1}{2}}= & \{\begin{array}{c} {\left[\frac{1}{4}\left[\frac{1}{{\left(\mu \right)}_{i+\frac{1}{2},j,k}}+\frac{1}{{\left(\mu \right)}_{i+\frac{1}{2},j+1,k}}+\frac{1}{{\left(\mu \right)}_{i+\frac{1}{2},j,k+1}}+\frac{1}{{\left(\mu \right)}_{i+\frac{1}{2},j+1,k+1}}\right]\right]}^{-1},\\ 0, \end{array}\\ & \{\begin{array}{c} if\;{\left(\mu \right)}_{i+\frac{1}{2},j,k},{\left(\mu \right)}_{i+\frac{1}{2},j+1,k},{\left(\mu \right)}_{i+\frac{1}{2},j,k+1},{\left(\mu \right)}_{i+\frac{1}{2},j+1,k+1}\neq 0\\ otherwise \end{array} \end{array}$$

where $\rho_{i}\left(i=r,\theta ,z\right)$ denotes the differential density parameter on a staggered grid of three velocity components, $\mu_{ij}\left(i,j=r,\theta ,z\right)$ represents the differential shear modulus parameter on a staggered grid of three shear stress components, and *i*, *j* and *k* denote the grid point numbers in the *r*, *θ*, and *z* directions, respectively.

### 2.3. Imaging Condition

The RTM is based on the 2-path wave formula, with time-reversed extrapolation conducted along the timeline. This process involves three key steps: (i) forward time propagation from the origin, (ii) propagation of the time-reversed distribution wave field recorded at acquired coordinates, and (iii) representation through cross-correlation. In the realm of pre-stack RTM imaging, various imaging conditions are commonly employed [52]. Herein, we employed cross-correlation representation conditions, continue with origin normalization, as follows:

(8) $$Image\left(x\right)=\sum_{t=0}^{t_{\max}}S\left(x,t\right)R\left(x,t\right)$$

where $x={\left[r,\theta ,z\right]}^{T}$ denotes the location, Image(x,t) represents the image result, and S(x,t) and R(x,t) represent the source field and the field at the receiver, respectively. The interpretations of the RTM imaging data primarily rely on the presence or absence of positive or negative image values. The unit of imaging results is the squared amplitude of the wavefield, which lacks physical significance in practical interpretations.

### 2.4. RTM Artifacts

**Figure 3:**
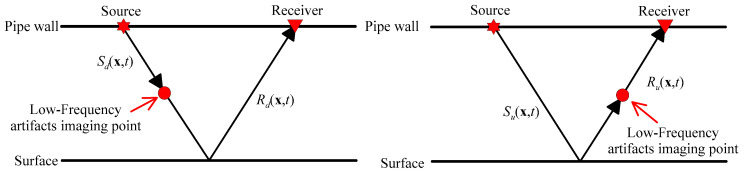
Schematic diagram of wave paths for low frequency artifacts.

The RTM can effectively image complex structures, but it will produce low-frequency artifacts in the application of imaging conditions. For the source field S(x,t) in Equation (8), it can be separated into upgoing waves $S_{u}\left(x,t\right)$ and down-going waves $S_{d}\left(x,t\right)$. In addition, the receiver field R(x,t) can be separated into upgoing waves $R_{u}\left(x,t\right)$ and downgoing waves $R_{d}\left(x,t\right)$. Figure 3 indicates the reasons for the generation of low-frequency artifacts. According to the cross-correlation imaging conditions, the cross-correlation between the downgoing waves $S_{d}\left(x,t\right)$ and $R_{d}\left(x,t\right)$ and the upgoing waves $S_{u}\left(x,t\right)$ and $R_{u}\left(x,t\right)$ will produce low-frequency artifacts [53].

Strong low-frequency artifacts can even interfere with effective signals and affect the imaging and interpretation of actual data. Hence, it becomes crucial to mitigate these arti-facts effectively in order to achieve high-quality images. The suppression of low-frequency artifacts can be performed with three aspects: (i) changing the propagation operator; (ii) modifying the imaging conditions; (iii) filtering methods after imaging. In this paper, we use Laplace filtering after imaging to suppress low-frequency artifacts, and the formula is given by [54]:

(9) $$\nabla^{2}=\frac{\partial^{2}}{\partial r^{2}}+\frac{\partial^{2}}{\partial \theta^{2}}+\frac{\partial^{2}}{\partial z^{2}}=-\frac{4\omega^{2}\cos^{2}\alpha }{v^{2}}$$

where α denotes the angle of incidence and v represents the velocity of the medium. $\nabla^{2}$ denotes the Laplace operator and ω represents the frequency. Laplace filtering is performed to multiply the filter factor $\cos^{2}\alpha$ in the imaging result, which is similar to the filtering in the angle domain. This filter factor will completely eliminate the noise in the imaging where the incident angle is close to 90°, while other parts with incident angles less than 90° can be suppressed.

### 2.5. Implementation

**Figure 4:**
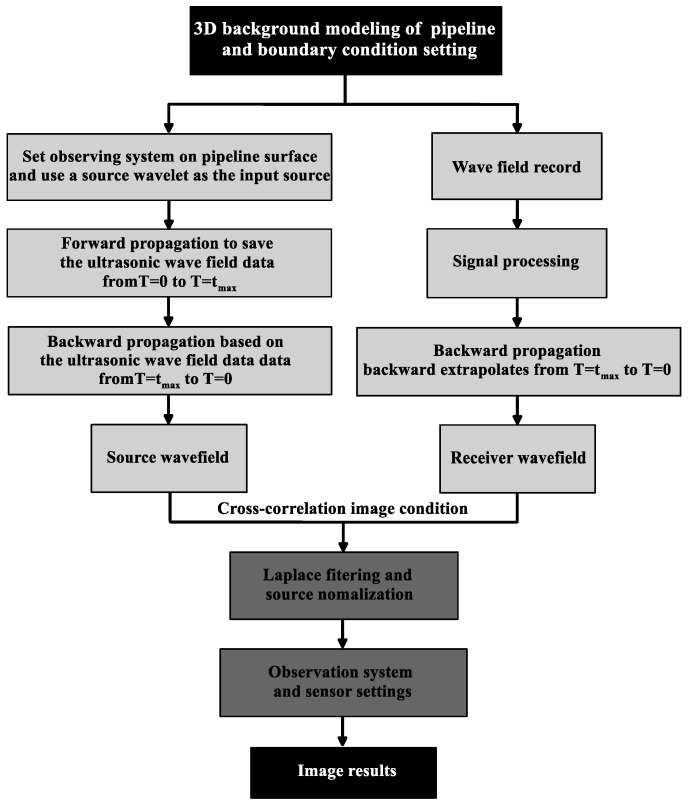
The implement flow figure.

Figure 4 depicts the sequential stages of the proposed method, with the initial step involving the construction of a 3D geophysical model of pressure pipelines and setting the boundary conditions reasonably. Then, a 3D ultrasonic wave field extrapolation simulation and RTM imaging of the pressure pipelines is conducted. The RTM imaging mainly includes three steps: (i) Set observing system on pipeline surface and use a source wavelet as the input source. Forward propagation to save the ultrasonic wave field data from T = 0 to T = t_max_. (ii) To acquire the ultrasonic source wave field data from T = t_max_ to T = 0, the recorded signal at the boundaries is subjected to time-reversed extrapolation and concurrently propagated back into the simulation domain. (iii) Three-dimensional imaging using cross-correlation condition of receiver and source wavefields. In the next step, the 3D imaging results were performed with Laplace filtering and source normalization. Finally, research on the observation system and sensor settings is carried out so that the final imaging result can be obtained by adopting the most reasonable scheme.

## 3. Numerical Simulation Results and Discussion

### 3.1. Survey Design and Modelling

**Figure 5:**
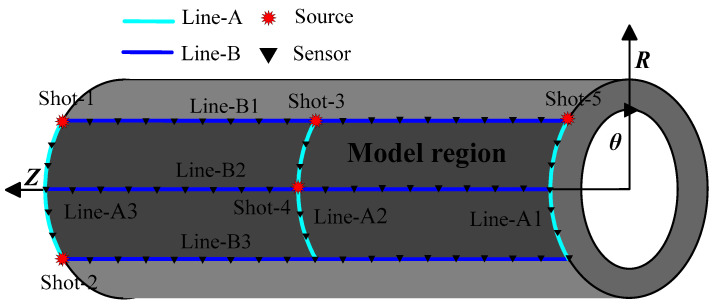
Schematic illustration of the observation system in UT.

The 3D cylindrical model of the pressure pipelines and the observation system are shown in Figure 5. The model region is covered by six survey lines. There are sensors located at the top, middle, and bottom of the exterior wall of the pipeline. It is designed to include three horizontal survey lines (cyan color) with sensor intervals of 1.0 mm (Line-A1, -A2, -A3) and three axial survey lines (blue color) with sensor intervals of 1.0 mm (Line-B1, -B2, -B3). The origin is positioned at the focal point and around the pipeline’s exterior wall (Shot-1~Shot-5).

**Figure 6:**
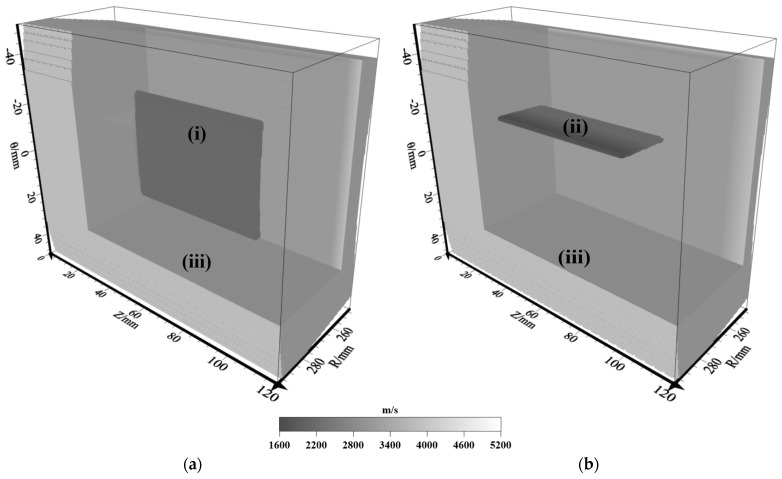
Three-dimensional pressure pipeline models. (**a**) Delamination model; (**b**) slag inclusion model. All parameters (i)–(iii) in the figure are shown in Table 1.

To assess the impact of sensor settings and different inner defects (e.g., slagging and delamination) on RTM imaging, two models were designed and are shown in Figure 6 where the model is indicated by the P wave velocity. These models are employed to validate the efficiency of the intended numerical modeling approach and explore the wave-field feature across various model scenarios. Model-1 was developed to assess the impact of the sensor settings and delamination defects (thickness = 2.0 mm, width = 51.45 mm and length = 60.0 mm) on RTM imaging. Model-2 is a slag inclusion (thickness = 2.0 mm, slag width = 22.5 mm and length = 60.0 mm), which is proposed for analyzing the RTM images of the slag inclusion defect. Table 1 provides the model factors.

In the wavefield extrapolation simulation, we used a staggered-grid TFDM approach that is spatially fourth-order and temporally second-order variable. We have designed a 3D cylindrical pressure pipeline model with a wall thickness of 45.0 mm, an inner diameter of 250.0 mm, a curve length of 102.97 mm (circular angle = 20°), and a longitudinal dimension of 120.0 mm. In the r-, θ-, and z-directions, the model dimensions are 120.0 mm × 102.97 mm (20°) × 45.0 mm. The circular and axial steps in the z-direction are both Δr = Δz = 0.2 mm. In the θ-direction, the angular increment is Δθ = 0.046°, and the longitudinal dimension rises with the wideness (0.2 mm and 0.236 mm at the interior and outer walls). The time sampling interval Δt is 0.01 µs. The source wavelet refers to a 0.7 Mhz ricker wavelet and a 0.33–1.16 Mhz signal bandwidth (−6 dB).

### 3.2. Wavefield Extrapolation Results

#### 3.2.1. Model-1

**Figure 7:**
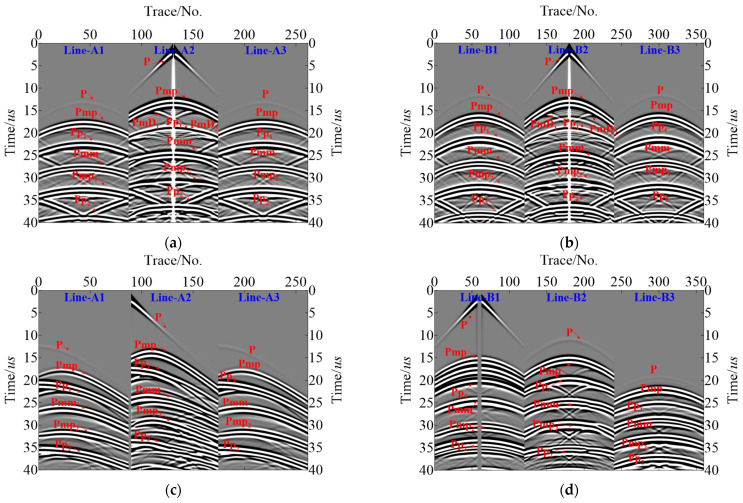
Simulated R-direction data of Model-1 (**a**) Line-A1~A3 of shot-4; (**b**) Line-B1~B3 of shot-4; (**c**) Line-A1~A3 of shot-3; (**d**) Line-B1~B3 of shot-3.

Using the TFDM with the cylindrical coordinates and corresponding boundary conditions proposed above, we successfully generated wave-field records for the dual survey lines via forward modeling based on the sensor settings and observation system shown in Figure 5. Full wave-field simulation according to pressure pipelines of Model-1 can be realized through these records, including direct P wave, reflected Pp wave, reflected Pmp wave, reflected Pmm wave, and diffracted PmD wave (Figure 7). Figure 7a,b are the records of shot-4 and Figure 7c,d are the records of shot-3. Trace indicates the number of the sensors in the survey line. It is observed that the energy of the direct P wave decreases with an increase in propagation distance, and the corresponding energy obtained from the sensors on both sides is very low, while the effective signal of the primary secondary Pmp generated by the delamination defects as well as the diffraction waves PmD1 and PmD2 generated from the delamination defect endpoints exhibit high energies, allowing for precise identification. Additionally, the reflected Pmm waves reflected from the inner wall of the pressure pipeline by delamination are also clearly visible, which has helped in imaging lower boundaries of delamination defects.

#### 3.2.2. Model-2

**Figure 8:**
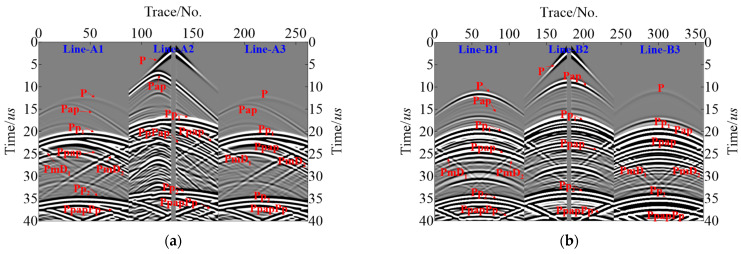
Simulated R-direction data for Model-2 (**a**) Line-A1~A3 for shot-4; (**b**) Line-B1~B3 for shot-4.

The sensor settings in Model-2 align well with those in Model-1, Figure 8a,b are the records of shot-4. As can be seen in Figure 8, the energy of the direct P wave in Line-A1, -A3, and -B3 is relatively weak, while the energy of the direct wave in Line-B1, -B2, and -A2 is relatively strong due to the relationship between the shot point’s location and the sensor design. The effective signals of Pap from the inclusion defects are weak in Line-θ1 and -θ3, but stronger in Line-θ2, which can clearly be identified. Furthermore, Ppap from the pipeline’s inner wall via the slag inclusion as well as PmD1 and PmD2 generated from the inclusion defects endpoints are clearly found in all the records, which has helped to image lower boundaries of slag inclusion.

### 3.3. RTM Imaging Artifacts Suppression

**Figure 9:**
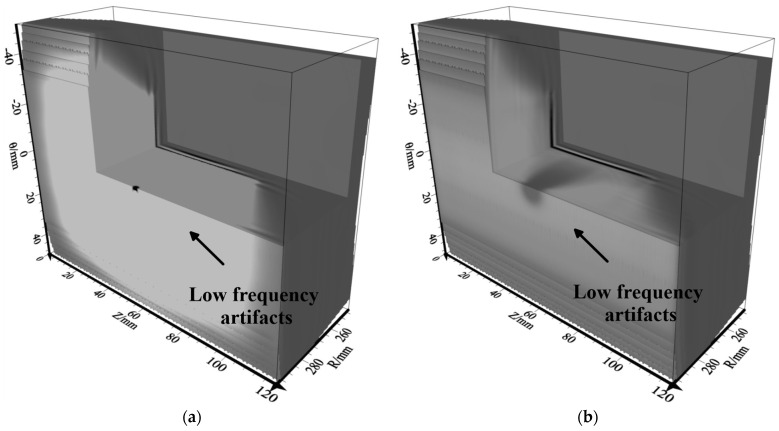

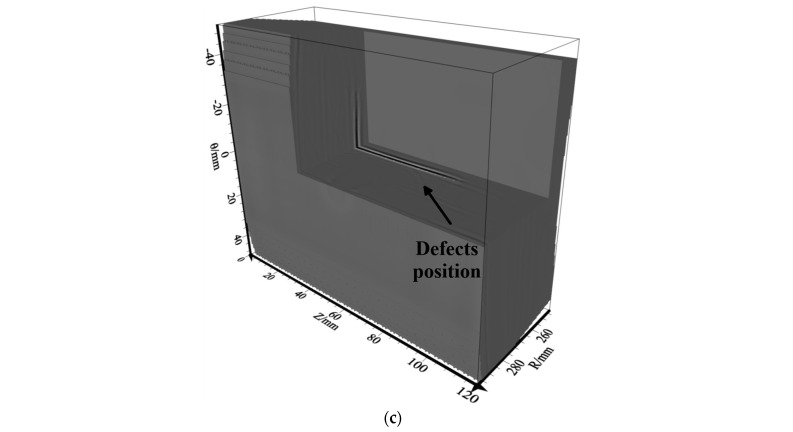
Imaging before and after artifacts suppressed. (**a**) Raw RTM representations; (**b**) imaging results of source illumination compensation processing; (**c**) imaging results of source illumination compensation and Laplace filtering processing.

RTM imaging will produce low frequency artifacts, which can suppress effective signals and affect the imaging and interpretation of actual data. Therefore, we use Laplace filtering and source illumination compensation to obtain the final image. As shown in Figure 9a, when imaging without processing there are serious low-frequency artifacts in the imaging results, which obviously interfere with the imaging of the slag inclusions. After the source illumination compensation is used, as can be seen in the Figure 9b, the low-frequency noise energy is attenuated to a certain extent, and the energy of the slag is strengthened, although it still affects the imaging results. Therefore, the Laplace method is further used for secondary processing. As depicted in Figure 9c, the low-frequency artifacts are well suppressed, and the resulting imaging has a high signal-to-noise ratio (SNR).

### 3.4. Sensor Settings and Observation System Discussion

**Figure 10:**
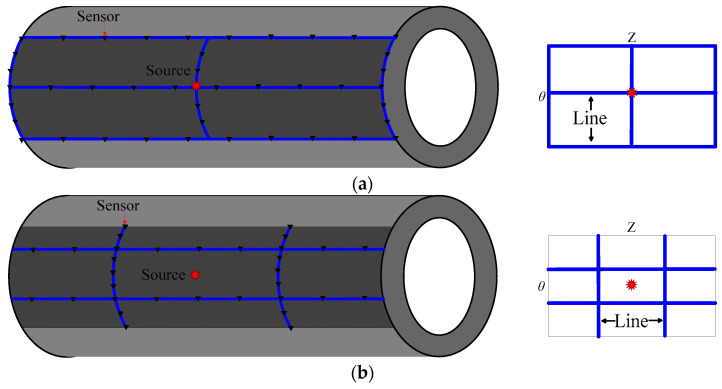

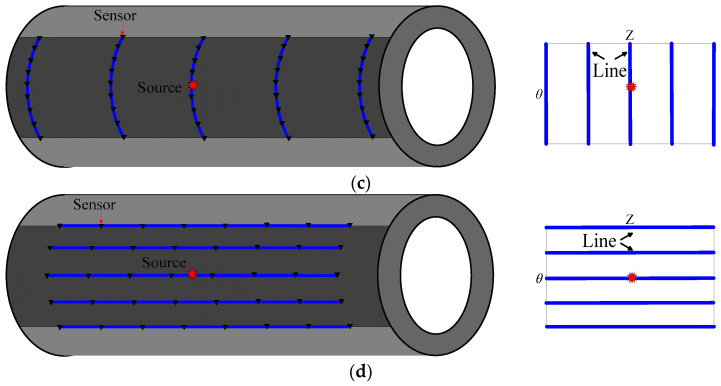
Schematic diagram of different sensor settings for different observation systems. (**a**) Three horizontal survey lines and three vertical survey lines; (**b**) two horizontal survey lines and two vertical survey lines; (**c**) five vertical survey lines; (**d**) five horizontal survey lines.

In the actual UT of pressure pipelines, due to the limitations of the site factors, the sensors and survey lines cannot be arranged arbitrarily. In order to detect the pipeline with the least number of survey lines, it is necessary to discuss in detail the observation system with the best sensor settings. To improve the imaging quality and investigate the influence of the sensor settings on the imaging results, we used different sensor arrangements which are located on the outside of the pipelines. As shown in Figure 10, there are four different sensor settings for different observation systems.

**Figure 11:**
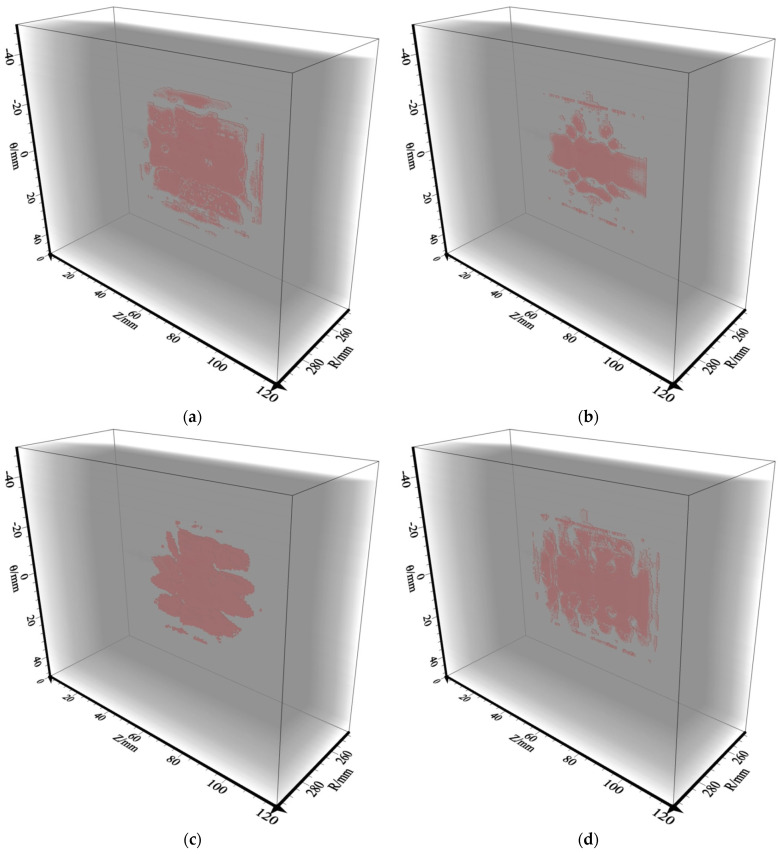
RTM imaging results for different sensor settings under single-shot (shot-4). (**a**) Three horizontal survey lines and three vertical survey lines imaging results corresponding to Figure 10a; (**b**) two horizontal survey lines and two vertical survey lines imaging results corresponding to Figure 10b; (**c**) five vertical survey lines imaging results corresponding to Figure 10c; (**d**) five horizontal survey lines imaging results corresponding to Figure 10d.

Figure 11 shows the RTM imaging results for different sensor settings. Figure 11a–d, respectively, correspond to the RTM imaging results for the four different sensor settings shown in Figure 10a–d. It can be clearly seen in Figure 11a that the delamination defect interface can be well presented in the first group of imaging results. Although the boundary is not very obvious, the position of the event corresponds to the position of the delamination defect. The imaging range accuracy up to 73.16%. In the imaging of the second group (Figure 11b), only part of the delamination defect can be imaged, and the defect structure cannot be accurately obtained, which does not meet the imaging requirements. The imaging range accuracy is only 49.35%. Figure 11c shows that the effective coverage of the interface area decreases in the third group, which is not conducive to the interpretation of the defect boundary structure, and only the defect structure in the middle region of the model can be analyzed and explained. The imaging range accuracy is 56.63%. As shown in Figure 11d, the fourth group has better imaging results in the region of defects and the obtained events are continuous, which reflects the information of the delamination defects well. The imaging range accuracy up to 79.76%. Therefore, it is shown that the single-shot observation system under the sensor settings of six survey lines (three horizontal and three vertical lines) or five horizontal survey lines can obtain better imaging results when performing RTM imaging of the delamination defects. This observation method is effective and feasible.

**Figure 12:**
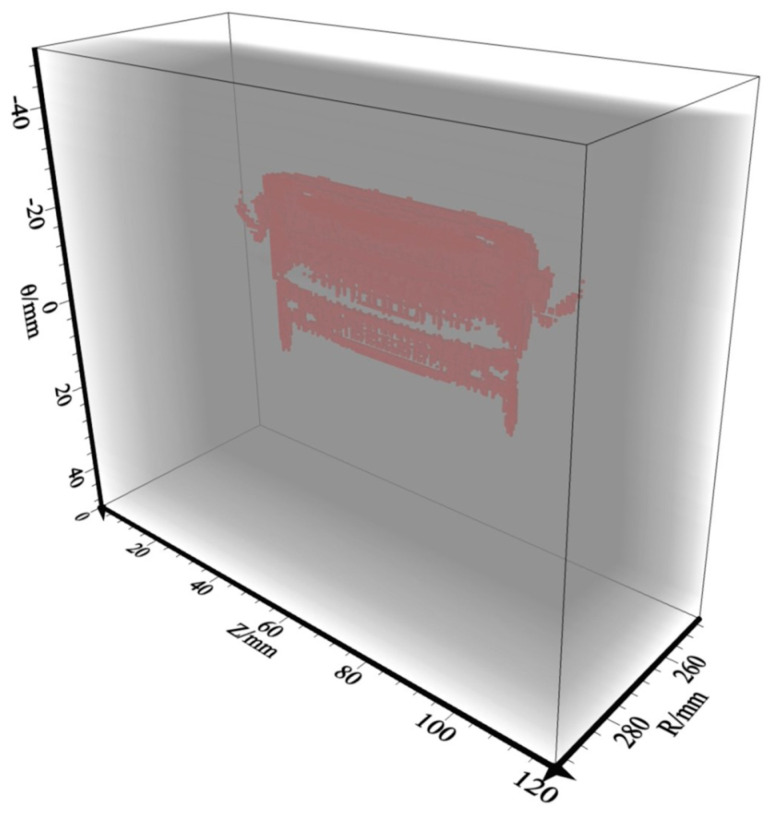
RTM imaging results under single-shot (shot-3).

The delamination defect interface can be well presented in the imaging results (Figure 11a), but the boundary is not very obvious. This is due to the fact thatshot-4 is located at the center point, which means that there are fewer reflection points at the boundary and corners compared to the center area, making the imaging results incomplete at the boundary. In order to analyze and study the imaging effects of different shot points, we used the records obtained from shot-3 (Figure 7c,d) to perform RTM imaging. From Figure 12 it can be seen that because of the special position of shot-3, RTM imaging can clearly show the upper boundary of the delamination defects; however, due to the limited number of sensors and the lack of reflection points, the non-imaging area of the lower boundary increases. The imaging range accuracy is only 62.08%. Therefore, the imaging accuracy of RTM is not only related to the sensor settings, but also to the location of the shot point.

### 3.5. RTM Imaging Results

**Figure 13:**
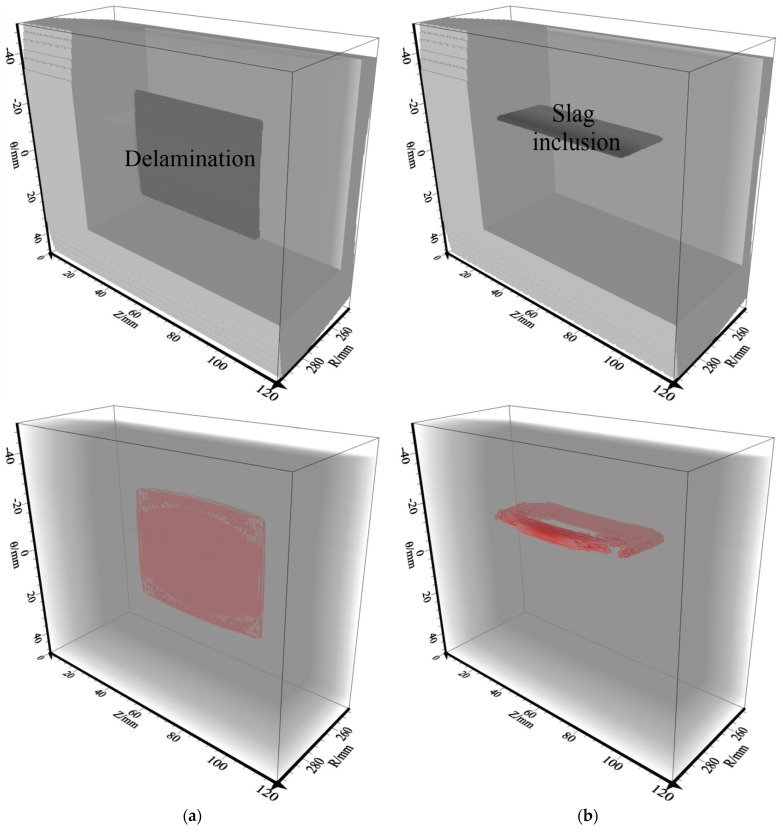
Five-shot stack results for 3D RTM imaging. (**a**) Delamination model; (**b**) slag inclusion model.

In order to obtain higher-precision imaging results and inspect the whole pipeline with the least cost (sensors), this paper adopts the method of multi-shot points (shot-1~shot-5) for stacking to obtain the final imaging result. The 3D ultrasonic RTM images were generated from a five-shot stack and denoised by source illumination compensation and Laplace filtering. Results for the delamination defect are shown in Figure 13a, it can clearly be seen that, when five shots are stacked, the imaging effect is evidently better than single-shot imaging, and the upper and lower interfaces of the delamination defects are clearly visible. There is a good match between the entire imaging result and Model-1 in terms of position and shape, and the defect information can be fully reflected in the interface region. The imaging range accuracy was up to 98.65%. It can be seen from Figure 13b that, for slag inclusions, the surrounding boundaries can be well imaged, especially the bottom boundary. While the absence of reflection points results in missing information in the central region, the whole imaging result matches well with Model-2 in terms of location, and the slag inclusion information can be fully reflected from the boundaries’ region. The imaging range accuracy is 63.44%.

## 4. Laboratory Experiment Results and Discussion

### 4.1. Specimens and Empirical Setup

**Figure 14:**
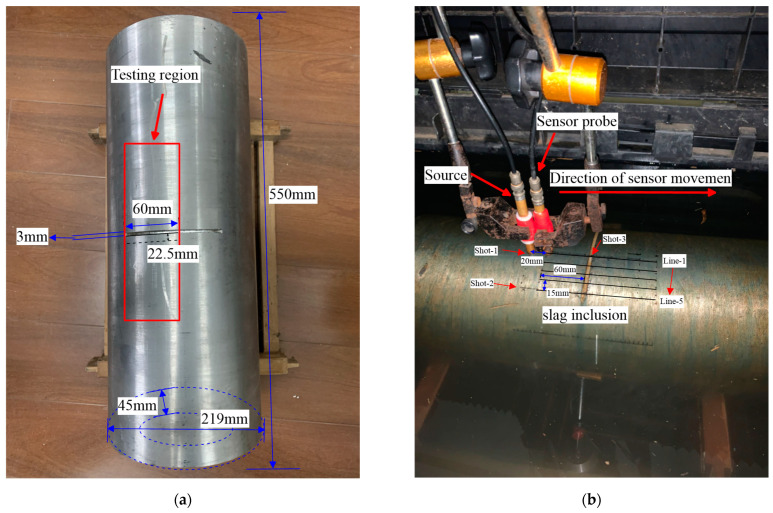

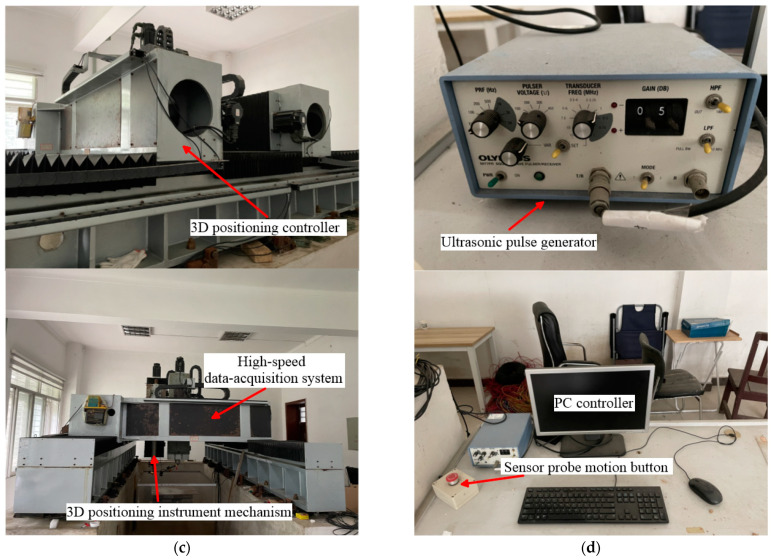
Specimens and ultrasonic experimental setup. (**a**) Specimen; (**b**) sensor settings and observing system. (**c**) Three-dimesional positioning instrument mechanism and high-speed data-acquisition system; (**d**) ultrasonic pulse generator and PC controller.

To validate the practicality of our proposed RTM method, we utilize field data acquired collected by lab equipment of ultrasonic seismic physics simulation in pressure pipelines with internal defects to perform RTM 3D imaging. The experimental specimen in this paper is the pressure pipelines, of which the length, wall thickness, and outside diameter are 550 mm, 45 mm, and 219 mm, respectively, as is shown in Figure 14a. To comply with the slag inclusions in actual UT, we injected cement into the crack of the specimen and maintained it for 2 days to create the low-velocity body. On the pipeline’s outer wall, there is a slag inclusion measuring 60 mm, 3 mm, and 22.5 mm in length, depth, and width, respectively. In order to detect the pressure pipelines with the least number of survey lines, we use the best sensor settings and observation system outlined above. The observation system is illustrated in Figure 14b; there are five horizontal survey lines in total, the distance between each line is 15 mm, and the line measures 150 mm in length. In addition, sensors are spaced 1 mm apart, and the sensors sample at a frequency of 50 MHz with a time range of 60 µs. The sources of three shots are located at endpoints and midpoints, respectively, the offset distance is 20 mm.

The lab equipment for the ultrasonic seismic physics simulation is illustrated in Figure 14c,d. The high-speed data acquisition system consists of a 3D positioning controller, a sensor probe, a computer controller, and an ultrasonic pulse transmitter. The experimental process is as follows: As a first step, the sampling parameters are set by the computer, including sampling frequency, time, length, and the start and end positions of the receiving sensor probe. Then, by pressing the sensor probe motion button, the 3D coordinate automatic positioning controller shifts within the sampling duration. When the ultrasonic receiving sensor arrives at the sampling point, the ultrasonic pulse generator immediately transmits a synchronization signal. At the end of the process, the ultrasonic signals captured by the ultrasonic receiving sensor are transmitted toward the computing device for further procedure.

### 4.2. Field Test Results

**Figure 15:**
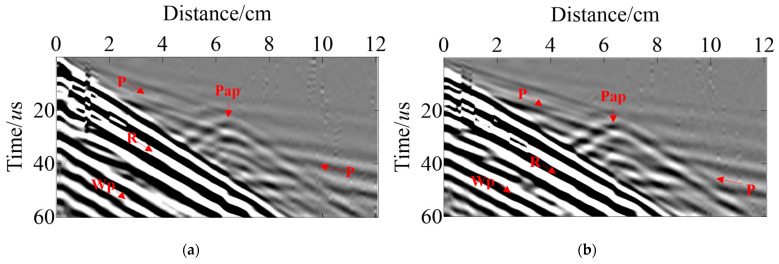

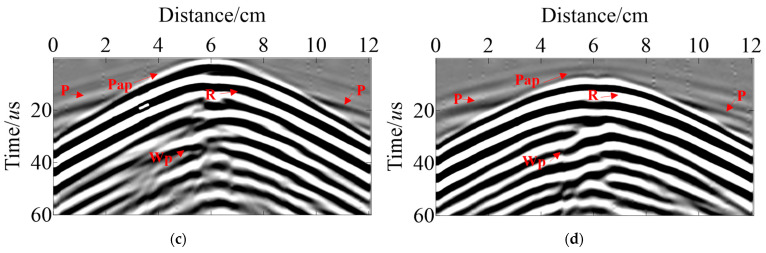
Field test data. The (**a**,**b**) is Line-1~Line-2 of shot-1; the (**c**,**d**) is Line-1~Line-2 of shot-3.

Figure 15 illustrates the actual data after it has been filtered from different shot points. The field test records include direct P wave, direct Wp wave, surface R wave, and diffracted Pap wave. The ultrasonic wave excites the direct Wp wave generated in water due to water being a coupling agent which travels slowly with high energy. In addition, the surface R wave energy is also higher, but the effective signal from the slag inclusion can still be identified efficiently and very clearly in Figure 15.

**Figure 16:**
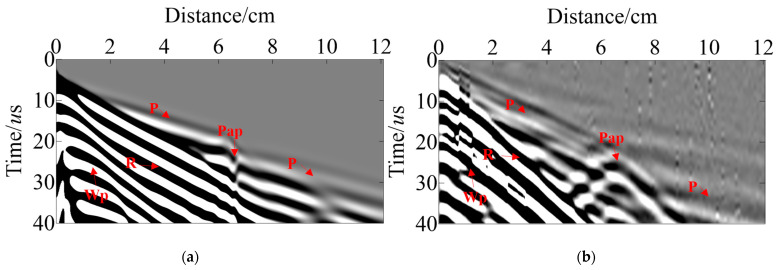
A comparison of the records between field tests and simulations. (**a**) Simulation record; (**b**) field test record.

According to the experimental specimens, we constructed a pressure pipeline model with the same slag inclusion, matching the physical parameters as well as the size. As can be seen in Figure 16, the actual excitation frequency is low due to the influence of noise and water layer absorption in the experiment. Although part of the effective wave field is covered by the surface R wave, the position of the slag inclusion effective signal diffracted Pap wave is the same in the simulation data and the experimental data. Thus confirming the reliability of our wave field extrapolation through TFDM simulation, and providing theoretical guidance for UT of pressure pipeline internal defects.

**Figure 17:**
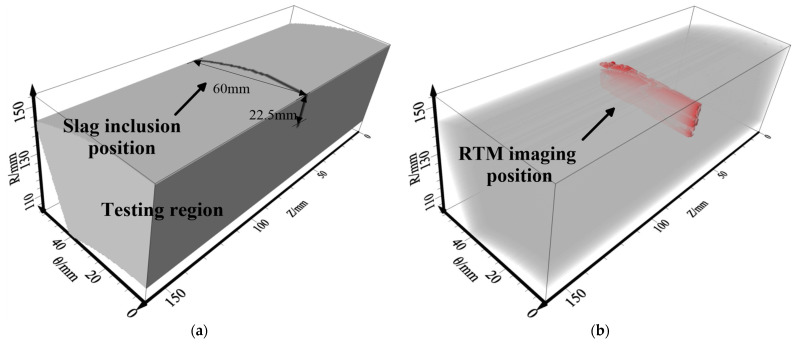
Results of the 3D RTM imaging field test. (**a**) Field test region model; (**b**) 3D RTM imaging.

On the basis of the best sensor settings and observation system above, the measured actual ultrasonic data of Line-1~Line-5 are used for 3D RTM imaging obtained by a three-shot stack. The image shown in Figure 17b is processed by Laplace filtering and source illumination compensation. The internal defects’ surrounding boundaries and lower interface can be well presented in the imaging results. There is a good match between the 3D imaging result and the experimental specimens in terms of position and shape. The imaging range accuracy was up to 97.85%. In addition, there is a high concentration of energy in the wave-field of the actual cement-filled defects in the concrete. The results of the field test confirm the reliability and feasibility of the 3D RTM method and the sensor settings proposed above for internal defect imaging of pressure pipelines.

## 5. Conclusions

Herein, we have proposed a 3D ultrasonic RTM imaging method to detect internal defects and sensor settings of various pressure pipelines. In order to match the natural cylindrical symmetry of pressure pipeline cavities, the algorithm is established in cylindrical coordinates. By setting the double free and absorbing boundary in cylindrical coordinates, the 3D elastic wave reverse time extrapolation of the pressure pipelines is simulated. For verification of the method’s accuracy and reliability, both numerical and experimental examples are presented. Using the 3D ultrasonic RTM method, we achieve higher-precision 3D imaging of the internal defects in the pressure pipelines through removing imaging artifacts. By comparing and analyzing the imaging results of different sensor settings, the design of the observation system is optimized to provide a basis for the imaging and interpretation of actual data. In contrast to traditional UT methods, the proposed 3D RTM method can obtain high-resolution, accurate 3D images of pipeline defects at the lowest cost. The improved accuracy can be attributed to the consideration of multi-wave reflection information from the bottom of the defect and the reflected wave from the inner wall of the pipe. Due to the optimal design of the sensors and observation system, the lowest cost is achieved. Numerical simulation and experiments are carried out to validate the proposed approach, and it has the potential to be applied in the practical UT of pressure pipelines.

## Figures and Tables

**Table 1:** Parameters of the two models.

Medium	Medium Number	$V_{p}\;\left(m/s\right)$	$\rho \;\left(kg/m^{3}\right)$
Delamination defects	(i)	1733.0	3300.0
Slag inclusion defects	(ii)	1600.0	2633.0
Pressure pipelines	(iii)	5200.0	7900.0

## Data Availability

The data presented in this study are available on request from the corresponding author.
